# *Plasmopara viticola* effector PvRXLR111 stabilizes VvWRKY40 to promote virulence

**DOI:** 10.1111/mpp.13020

**Published:** 2020-11-30

**Authors:** Tao Ma, Shuyun Chen, Jiaqi Liu, Peining Fu, Wei Wu, Shiren Song, Yu Gao, Wenxiu Ye, Jiang Lu

**Affiliations:** ^1^ Center for Viticulture and Enology School of Agriculture and Biology Shanghai Jiao Tong University Shanghai China

**Keywords:** cell death, *Plasmopara viticola*, RXLR effector, virulence, *WRKY40*

## Abstract

*Plasmopara viticola*, the causal organism of grapevine downy mildew, secretes a vast array of effectors to manipulate host immunity. Previously, several cell death‐inducing PvRXLR effectors have been identified, but their functions and host targets are poorly understood. Here, we investigated the role of PvRXLR111, a cell death‐inducing RXLR effector, in manipulating plant immunity. When coexpressed with other PvRXLR effectors, PvRXLR111‐induced cell death was prevented. Transient expression of PvRXLR111 in *Nicotiana benthamiana* suppressed bacterial flagellin peptide flg22‐elicited immune responses and enhanced *Phytophthora capsici* infection. PvRXLR111 induction in *Arabidopsis* increased susceptibility to *Hyaloperonospora arabidopsidis*. PvRXLR111 expression in *Pseudomonas syringae* promoted bacterial colonization. By immunoprecipitation‐mass spectrometry analysis, yeast two‐hybrid, pull‐down, and bimolecular fluorescence complementation assays, it was shown that PvRXLR111 interacted with *Vitis vinifera* putative WRKY transcription factor 40 (VvWRKY40), which increased VvWRKY40 stability. Transient expression of VvWRKY40 in *N. benthamiana* inhibited flg22‐induced reactive oxygen species burst and enhanced *P. capsici* infection and silencing *NbWRKY40* attenuated *P. capsici* colonization. These results suggest VvWRKY40 functions as a negative regulator in plant immunity and that PvRXLR111 suppresses host immunity by stabilizing VvWRKY40.

## Graphical abstract

PvRXLR111 from *Plasmopara viticola* suppresses PTI to promote pathogen virulence by stabilizing VvWRKY40, which functions as a negative regulator in plant immunity.

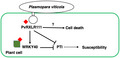

## 1 INTRODUCTION

Over time, plants have been equipped with two layers of immune system for survival (Jones & Dangl, 2006). The first layer, termed pattern‐triggered immunity (PTI), is triggered by the sensing of many conserved molecules from the pathogen/microbe‐associated molecular pattern (PAMP/MAMP), such as a flagellin peptide flg22 from bacteria and an elicitin INF1 from oomycetes (Ranf, 2017), through pattern recognition receptors, leading to a series of immune responses including reactive oxygen species (ROS) burst, callose deposition, and expression of plant defence genes (Li et al., 2016). For successful infection, pathogens deploy many effectors to suppress PTI. On the other hand, resistant plants have resistance (R) proteins to sense these effectors, which triggers the second layer of immunity known as effector‐triggered immunity (ETI). An important output of ETI is programmed cell death, which is thought to act against biotrophic pathogens by restricting pathogen access to water and nutrients (Cui et al., 2015; Glazebrook, 2005).

WRKY transcription factors are important regulators of plant defence responses (Birkenbihl et al., 2017a). WRKY40 has an impact on plant immunity, especially in PAMP‐triggered immunity (Pandey et al., 2010). The double mutants *wrky40 wrky18* enhanced *Arabidopsis* resistance to *Pseudomonas syringae* and *Golovinomyces orontii*, suggesting that AtWRKY40 negatively regulates plant defence (Shen et al., 2007; Xu et al., 2006). More recently, a positive role of WRKY40 from *Cicer arietinum* in plant resistance was reported (Chakraborty et al., 2019). However, the role of WRKY40 from grapevine in immunity remains unknown.

Many pathogenic oomycetes, such as *Phytophthora sojae*, *Phytophthora infestans*, and *Plasmopara halstedii*, consistently threaten agricultural production (Wang et al., 2019). The oomycete effectors are mainly classed as apoplastic and intracellular effectors (Kamoun, 2006). RXLR (Arg‐any amino acid‐Leu‐Arg) effectors and Crinkler (CRN) effectors are two major types of intracellular effectors whose major function is to subvert host immune responses such as ROS production and MAPK activation (Bozkurt et al., 2012; Wang et al., 2019). In terms of RXLR effectors, there is some evidence that RXLR effectors could also induce plant cell death upon recognition by corresponding R proteins or unknown mechanisms (Du et al., 2015; Huang et al., 2018). However, the biological significance of cell death‐inducing effectors in plant immunity remains largely unknown.

*Plasmopara viticola*, the causal organism of grapevine downy mildew, causes enormous economic loss to the grape industry (Gessler et al., 2011). Many RXLR effectors of *P. viticola* have been predicted by bioinformatics analysis of genomic or transcriptomic information (Brilli et al., 2018; Mestre et al., 2012; Yin et al., 2015, 2017). In our previous studies, 83 PvRXLR effectors, cloned from a Chinese *P. viticola* isolate, were characterized based on subcellular location and the ability to suppress cell death induced by two elicitors, INF1 and Bax (Liu et al., 2018). Many PvRXLR effectors were found to be able to suppress plant immunity, and of these PvRXLR131 was further shown to suppress plant immunity by targeting BKI1 (Lan et al., 2019). Meanwhile, several PvRXLRs were shown to induce cell death when expressed in *Nicotiana benthamiana* (Liu et al., 2018). However, the role of these PvRXLRs in manipulating plant immunity remains largely unknown. For example, PvRXLR111 was preliminarily characterized as an effector that induced cell death and possessed a functional signal peptide, which was expressed during *P. viticola* infection (Liu et al., 2018). In the present study, we further characterized the role of PvRXLR111 in plant–microbe interaction and identified VvWRKY40 as one of its host targets. The present results suggest that VvWRKY40 is a negative regulator in plant immunity and PvRXLR111 suppresses PTI via stabilizing VvWRKY40 to promote pathogen virulence.

## 2 RESULTS

### 2.1 PvRXLR111 suppresses flg22‐induced immune responses

**FIGURE 1:**
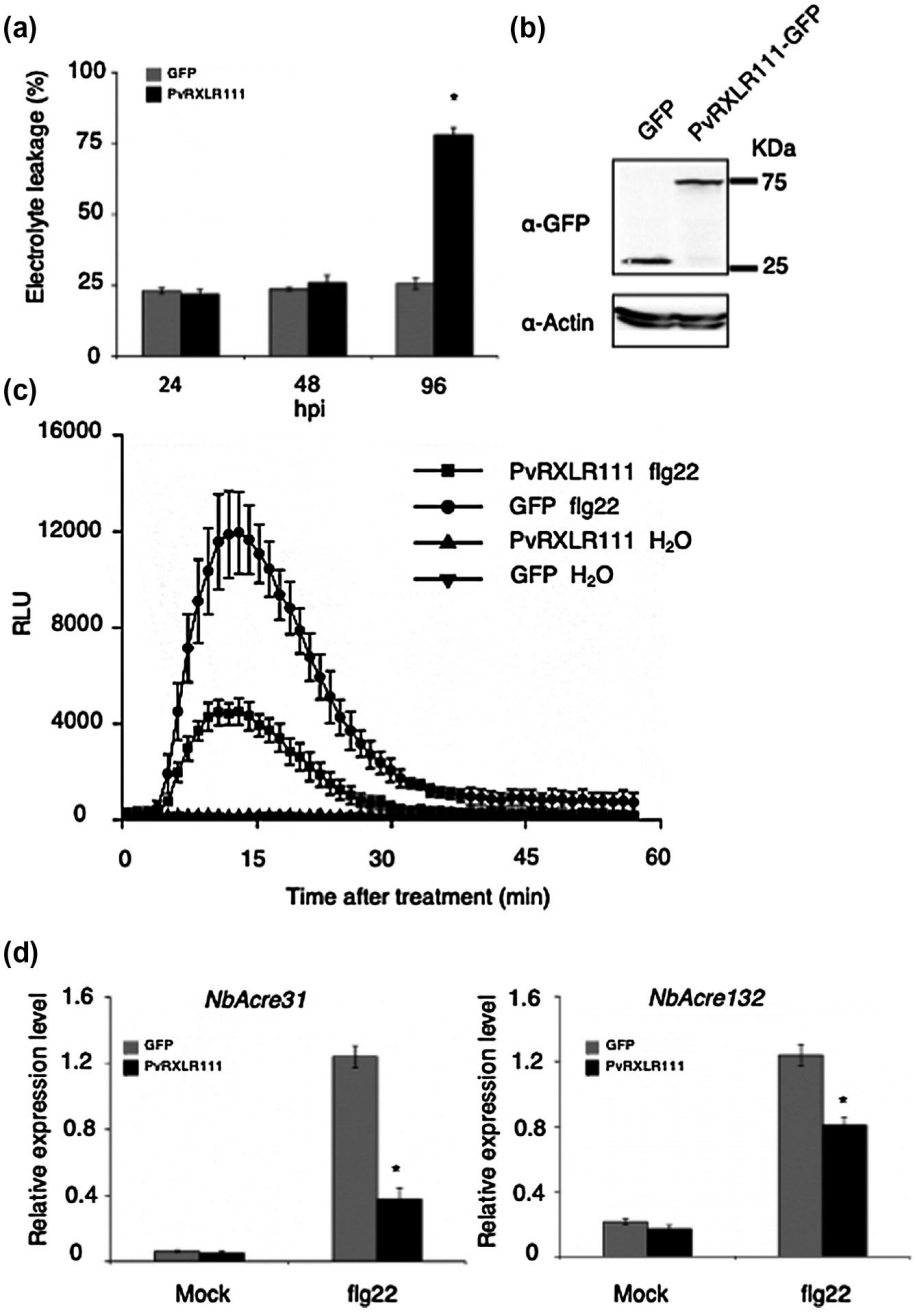
PvRXLR111 suppresses flg22‐induced immune responses. (a) Quantification of PvRXLR111‐induced cell death by electrolyte leakage measurement. Mean and standard deviations were calculated from three biological replicates (Student's *t* test, *p < .05). (b) Western blot analysis of proteins from *Nicotiana benthamiana* leaves transiently expressing green fluorescent protein (GFP) and PvRXLR111‐GFP at 24 hr postinfiltration (hpi). (c) PvRXLR111 inhibits flg22‐induced oxidative burst in *N. benthamiana*. Reactive oxygen species (ROS) production induced by 1 μM flg22 or water (mock) was determined in leaf discs of *N. benthamiana* transiently expressing either PvRXLR111‐GFP or GFP at 24 hpi. The results shown are representative of three independent experiments. Each data point consists of six replicates. Error bars indicate standard deviation. (d) The relative expression levels of *NbAcre31* and *NbAcre132* in *N. benthamiana* leaves transiently expressing PvRXLR111‐GFP or GFP at 1 hr after treatment with 1 μM flg22 or water were assayed by quantitative reverse transcription PCR. Mean and standard deviations were calculated from three biological replicates (Student's *t* test, *p < .05)

In our previous study, PvRXLR111, a 451 amino acid secreted protein with an RXLR motif, was shown to localize in the nucleus and could induce cell death in *N. benthamiana* (Liu et al., 2018). Our previous results do not exclude the possibility that PvRXLR111 also carries virulence functions. Although PAMPs are perceived by different receptors, downstream signalling events are largely shared, including ROS production (Couto & Zipfel, 2016; Ye & Murata, 2016). To investigate the role of PvRXLR111 in suppressing plant immunity, we checked the effect of PvRXLR111 on ROS production induced by the well‐studied MAMP, flg22, in *N. benthamiana*. Because long‐term expression of PvRXLR111 in *N. benthamiana* induces cell death (Liu et al., 2018), we chose to investigate at an early time point. As shown in Figure 1a,b, accumulation of PvRXLR111 protein was observed and did not induce cell death when expressed in *N. benthamiana* for 24 hr. On the other hand, the PvRXLR111 expression strongly suppressed flg22‐induced ROS production (Figure 1c). We also monitored the callose deposition and mRNA accumulation of two PTI marker genes (*Acre132* and *Acre31*) in leaf discs of *N. benthamiana* transiently expressing either green fluorescent protein (GFP) or PvRXLR111‐GFP after treatment with flg22. Compared with GFP transient expression, flg22‐induced callose deposition was significantly reduced in the area expressing PvRXLR111 (Figure S1). In both GFP‐ and PvRXLR111‐GFP‐expressing areas, a strong induction of *Acre132* and *Acre31* was observed as compared to mock treatment. However, the expression level of both *Acre132* and *Acre31* was significantly lower in the PvRXLR111‐expressing zone than in the control (Figure 1d). These results suggest that PvRXLR111 suppresses PTI responses.

### 2.2 PvRXLR111 promotes pathogen colonization

**FIGURE 2:**
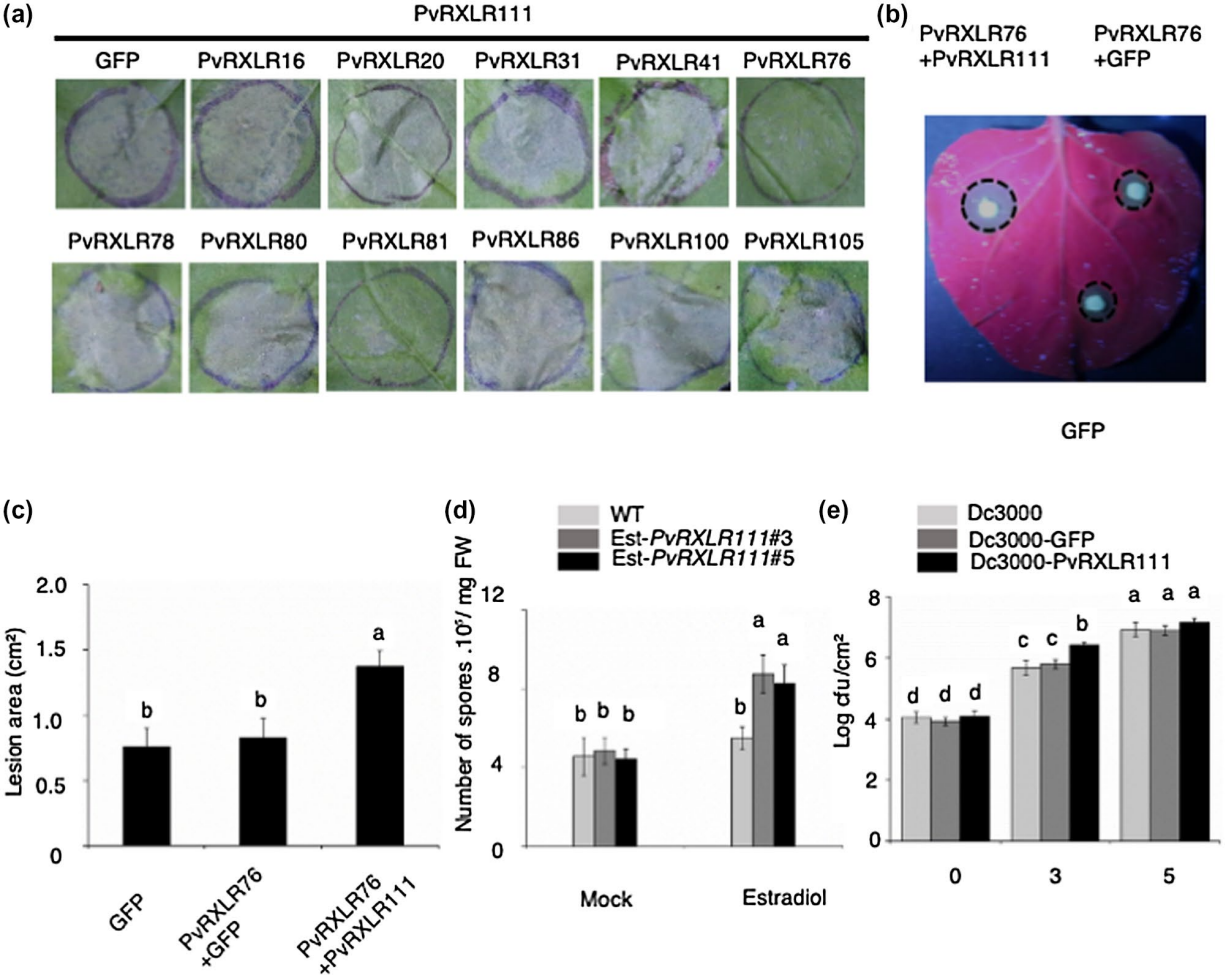
PvRXLR111 promotes the growth of pathogens. (a) PvRXLR111‐induced cell death could be suppressed by other PvRXLR effectors. *Agrobacterium tumefaciens* cells carrying constructs encoding the indicated PvRXLR effector or green fluorescent protein (GFP) with PvRXLR111 were coinfiltrated in *Nicotiana benthamiana*. The pictures were taken 5 days postinfiltration. (b) and (c) Transient expression of PvRXLR111 enhances *Phytophthora capsici* colonization. The infiltrated area coexpressing PvRXLR76 with GFP or PvRXL111 was inoculated with *P. capsici* at 24 hr postinfiltration. Photograph (b) was taken under UV light at 54 hr postinoculation and the lesion area was measured at the same time point. The lesion area (c) was calculated from three independent biological replicates. Different letters indicate significantly different values at *p* < .05 (one‐way analysis of variance, [ANOVA], Tukey's HSD test). (d) Two‐week‐old transgenic lines 24 hr after spray treatment with estradiol or water were inoculated with *Hyaloperonospora arabidopsidis* Noco2. Spores were counted at 5 days postinoculation (dpi). Data are means ± *SE* from three biological replicates. Different letters indicate significantly different values at *p* < .05 (one‐way ANOVA, Tukey's HSD). (e) PvRXLR111 promotes the growth of *Pseudomonas syringae* pv. *tomato* DC3000. Pst DC3000 carrying GFP‐His or PvRXLR111‐His was infiltrated into *Arabidopsis* leaves at OD_600_ = 0.001. Bacterial populations were determined at 0, 3, and 5 days after inoculation. Mean and standard deviations were calculated from three biological replicates. Different letters indicate significantly different values at *p* < .05 (one‐way ANOVA, Tukey's HSD)

The PTI suppression of PvRXLR111 drove us to further study the role of PvRXLR111 in plant–pathogen interaction. Because it is difficult to genetically modify *P. viticola* and grapevine, the role of PvRXLR111 in plant–pathogen interaction was investigated using the *N. benthamiana–Phytophthora capsici* pathosystem instead. Previous results have shown that the long‐term effect of cell death‐inducing RXLR effectors on plant immunity can be studied in the presence of other RXLR effectors that inhibit cell death (Wang et al., 2011). Our previous study also found that immunity triggered by PvRXLR16, a cell death‐inducing effector, could be suppressed by other PvRXLR effectors (Xiang et al., 2017). Therefore, we were interested in searching for *P. viticola* effectors, if there are any, that could suppress PvRXLR111‐triggered cell death. Based on different abilities to suppress plant immunity discovered in our laboratory (Liu et al., 2018), 11 PvRXLRs were chosen to conduct an *Agrobacterium* coinfiltration experiment in this study. As shown in Figures 2a and S2a, among the 11 PvRXLRs investigated, PvRXLR76 and PvRXLR81 strongly suppressed PvRXLR111‐induced cell death when coexpressed in *N. benthamiana*, with PvRXLR76 having the strongest inhibitory effect. We investigated the role of PvRXLR111 in the *N. benthamiana–P. capsici* interaction in the presence of PvRXLR76. PvRXLR111 was coexpressed with PvRXLR76 in *N. benthamiana* and then the infiltrated area was inoculated with *P. capsici*. The area expressing PvRXLR111 exhibited a larger lesion area, indicating that *N. benthamiana* resistance to *P. capsici* was compromised by PvRXLR111 (Figure 2b,c).

To further evaluate the contribution of PvRXLR111 in the *Arabidopsis–Hyaloperonospora arabidopsidis* pathosystem, we constructed transgenic *Arabidopsis* lines expressing PvRXLR111 under an estradiol‐inducible promoter. We tested to see if PvRXLR111 induction in *Arabidopsis* could reduce resistance to *H. arabidopsidis*. As shown in Figure 2d, estradiol treatment of ED‐PvRXLR111 plants but not mock treatment showed increased susceptibility to *H. arabidopsidis* Noco2. The expression of PvRXLR111 in transgenic *Arabidopsis* lines was confirmed by western blot (Figure S3a). We also investigated the role of PvRXLR111 in plant–pathogen interactions using the *Arabidopsis–Pseudomonas syringae* pathosystem. We generated *P. syringae* pv. *tomato* DC3000 (Pst DC3000) expressing PvRXLR111 driven by the *AvrPto* promoter to investigate whether it enhances Pst DC3000 infection. The expression of PvRXLR111 in Pst DC3000 was confirmed by western blot (Figure S3b). The wild‐type *Arabidopsis* plants were inoculated with Pst DC3000, Pst DC3000 (GFP‐His), and Pst DC3000 (PvRXLR111‐His) to determine the growth of bacteria. Compared with the control, the bacteria expressing PvRXLR111 exhibited a significantly higher population size at 3 days postinoculation (dpi), although a similar bacterial titre was observed at 5 dpi, suggesting a contribution of PvRXLR111 to virulence (Figure 2e). These results suggest that pathogens can use PvRXLR111 to promote pathogen colonization.

### 2.3 PvRXLR111 interacts with VvWRKY40

**FIGURE 3:**
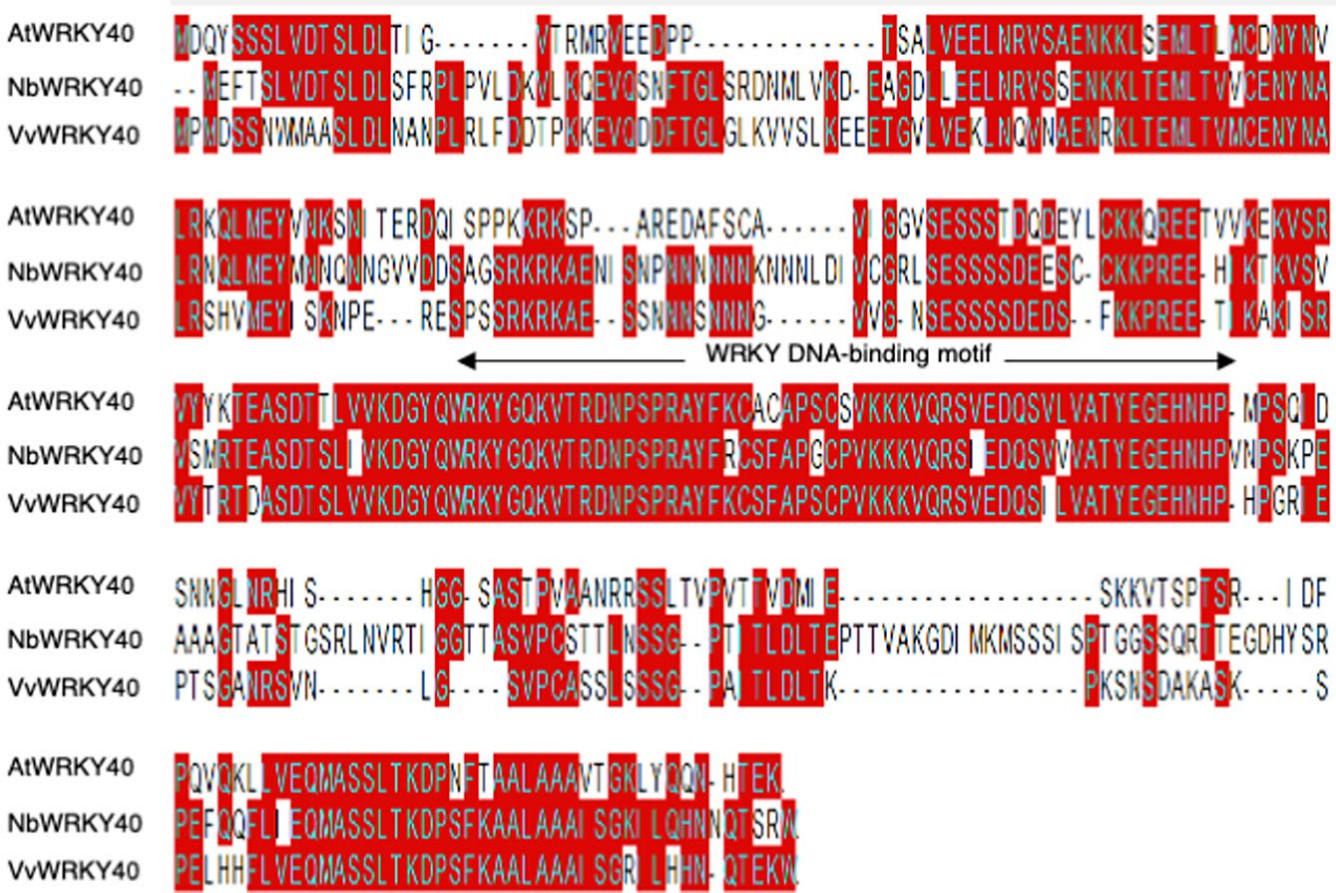
Sequence alignment of AtWRKY40, NbWRKY40, and VvWRKY40. Amino acids similar in the three proteins are indicated by red and the WRKY DNA‐binding motif is shown with an arrow

**FIGURE 4:**
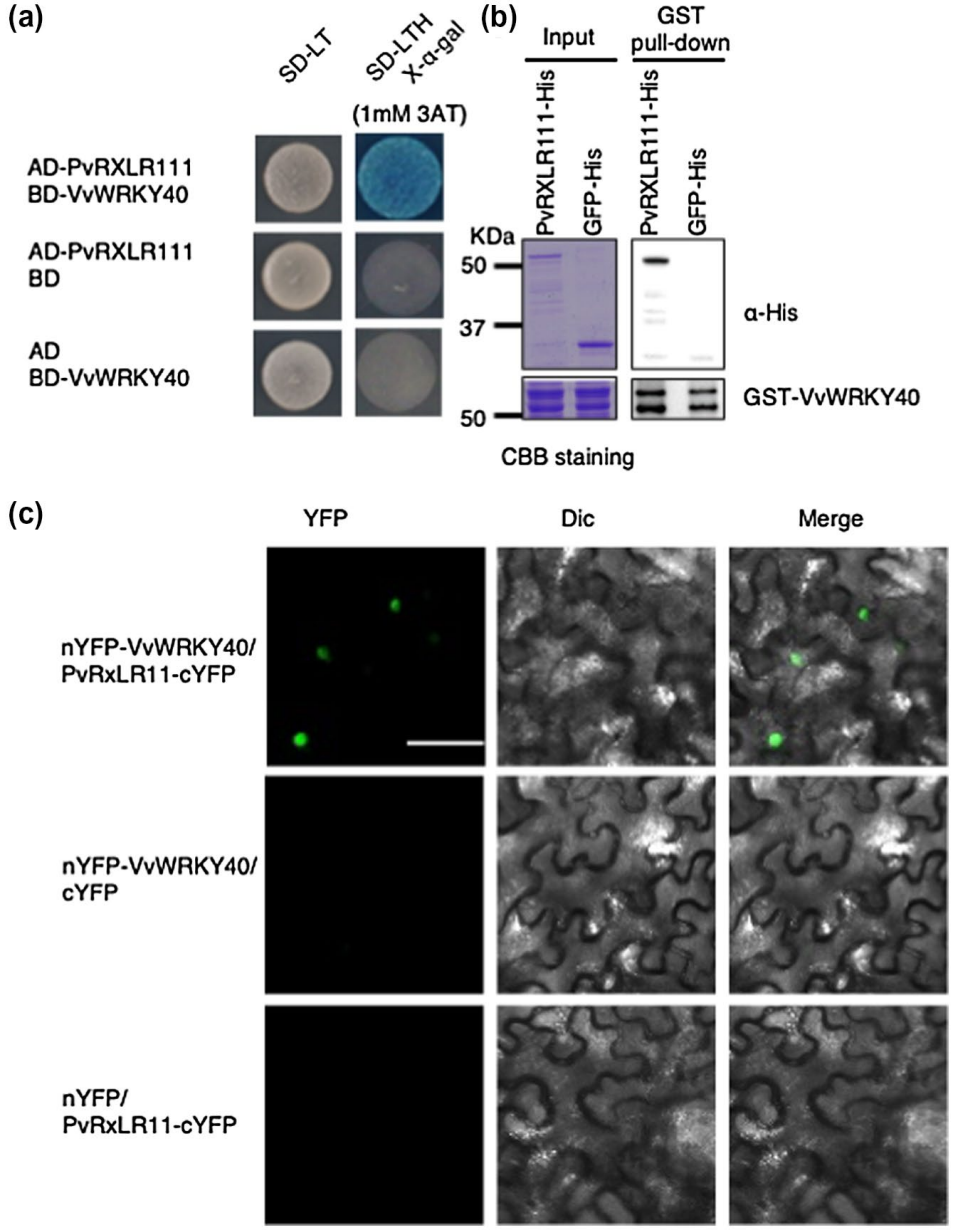
PvRXLR111 interacts with VvWRKY40. (a) PvRXLR111 interacts with VvWRKY40 in GAL4 yeast two‐hybrid system. Yeast cells coexpressing the indicated constructs were grown on nonselective SD−Trp−Leu medium or the selective medium SD−Trp−Leu−His containing X‐α‐gal with supplementary 1 mM 3AT. (b) In vitro pull‐down showing the interaction of PvRXLR111 with VvWRKY40. PvRXLR111‐His, GFP‐His, and GST‐VvWRKY40 were expressed in *Escherichia coli*. PvRXLR111‐His pulled down by GST‐VvWRKY40 was detected by western blotting using an anti‐His antibody. (c) Bimolecular fluorescence complementation assays showing the interaction of PvRXLR111 with VvWRKY40 in *Nicotiana benthamiana*. *Agrobacterium tumefaciens* cells carrying constructs encoding the indicated protein were coinfiltrated in *N. benthamiana*. Scale bars, 50 µm

We performed an immunoprecipitation‐mass spectrometry (IP‐MS) assay in *N. benthamiana* to identify the targets of PvRXLR111. We detected seven *N. benthamiana* proteins that possibly associated with PvRXLR111 (Table S1). Among them, NbWRKY40 received our attention as one of the strongest candidates due to the important role of WRKY transcription factors in plant defence responses (Birkenbihl et al., 2017a). As shown in Figure 3, VvWRKY40 shared the highest sequence identity with AtWRKY40 (54.7%), followed by NbWRKY40 (52.5%), which led us to investigate the interaction between PvRXLR111 and VvWRKY40 in vitro and in vivo by yeast two‐hybrid, pull‐down, and bimolecular fluorescence complementation (BiFC) assays. The coding sequences of *PvRXLR111* and *VvWRKY40* were fused with the GAL4 transcriptional activation domain (AD) and the GAL4 DNA‐binding domain (BD), respectively. Then, the constructs were transformed into the yeast strain AH109. As shown in Figure 4a, only the yeast strain transformed with AD‐*PvRXLR111* and BD‐*VvWRKY40* grew on the selection medium, indicating that PvRXLR111 interacts with VvWRKY40 in yeast. Furthermore, we performed a pull‐down assay using glutathione S‐transferase (GST)‐tagged VvWRKY40 and His‐tagged PvRXLR111 synthesized from *Escherichia coli*. As shown in Figure 4b, PvRXLR111‐His was pulled down by VvWRKY40‐GST compared with GFP‐His. We also conducted a BiFC assay to investigate the PvRXLR111–VvWRKY40 interaction in vivo. In contrast to the negative control, strong fluorescence was detected in the nucleus of *N. benthamiana* cells coexpressing nYFP‐VvWRKY40 and PvRXLR111‐cYFP, suggesting that PvRXLR111 interacts with VvWRKY40 in vivo (Figure 4c). These results indicate that PvRXLR111 interacts with VvWRKY40 in vitro and in vivo.

### 2.4 Overexpressing VvWRKY40 promotes, and silencing NbWRKY40 attenuates, *P. capsici* colonization

**FIGURE 5:**
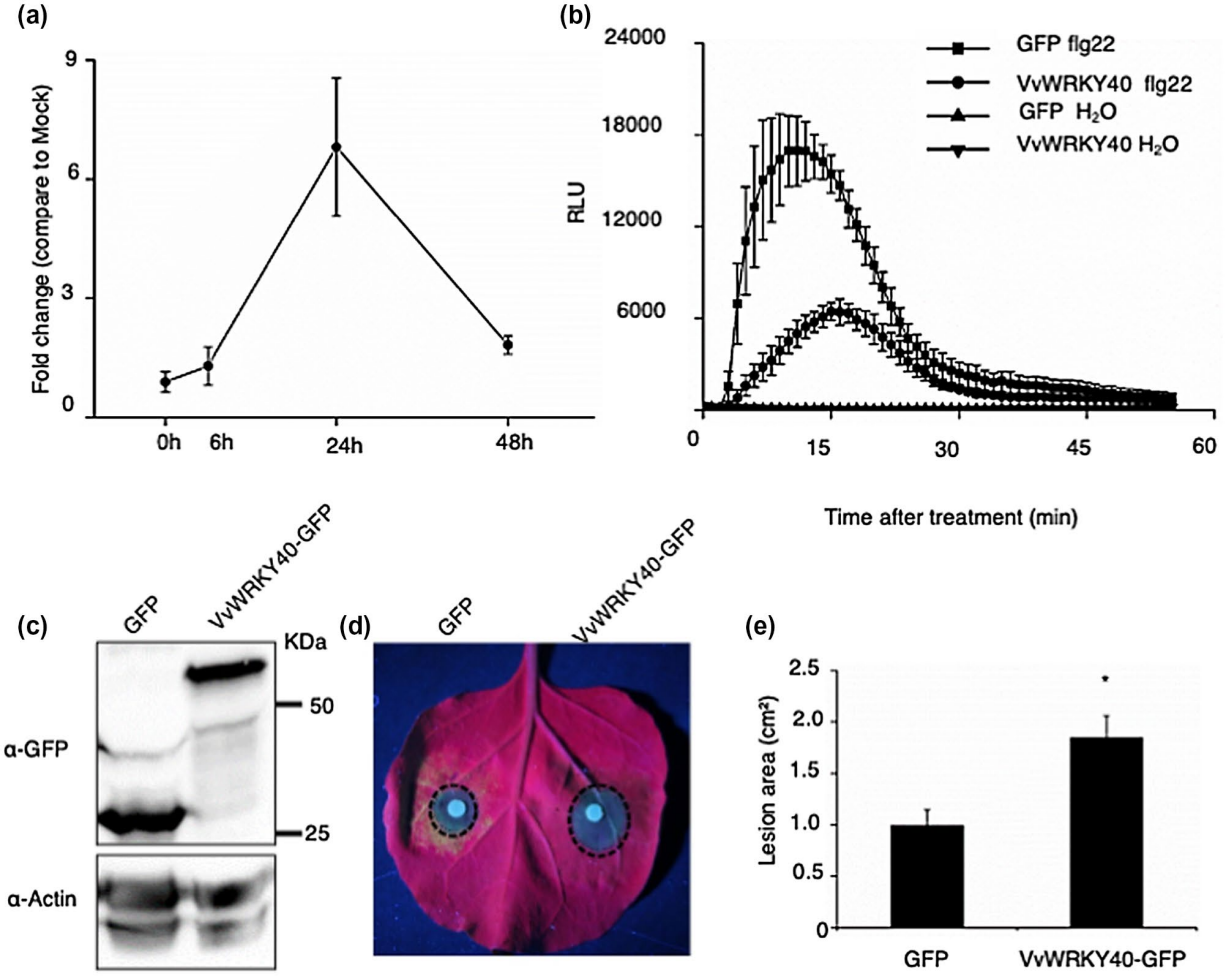
VvWRKY40 suppresses plant immunity. (a) Expression pattern of *VvWRKY40* during *Plasmopara viticola* infection in *Vitis vinifera*. Leaf disks of *V. vinifera* susceptible cultivar Thomson Seedless were inoculated with *P. viticola* for RNA isolation. The expression level of *VvWRKY40* was measured by quantitative reverse transcription PCR. (b) VvWRKY40 inhibits flg22‐induced oxidative burst in *Nicotiana benthamiana*. Reactive oxygen species (ROS) production induced by 1 μM flg22 or water (mock) was determined in leaf discs of *N. benthamiana* transiently expressing either green fluorescent protein (GFP) or VvWRKY40‐GFP at 48 hr postinoculation (hpi). The results shown are representative of three independent experiments. Each data point consists of six replicates. Error bars indicate standard deviation. (c) Western blot analysis of proteins from *N. benthamiana* leaves transiently expressing VvWRKY40‐GFP or GFP. (d) and (e) Transientexpression of VvWRKY40 enhances *Phytophthora capsici* colonization. The infiltrated area expressing GFP or VvWRKY40‐GFP was inoculated with *P. capsici* at 24 hpi. Photograph (d) was taken under UV light at 54 hpi and the lesion area was measured at the same time point. The lesion area (e) was calculated from three independent biological replicates using six leaves each. Error bars indicate standard deviation (Student's *t* test, *p < .05)

We analysed *VvWRKY40* expression during *P. viticola* infection in grape. The total RNA of inoculated leaf discs was isolated. The transcript level of *VvWRKY40* was induced by *P. viticola* infection, up‐regulated to a higher level at 24 hpi, and then decreased (Figure 5a), indicating that VvWRKY40 is involved in host–pathogen interaction. To investigate the function of VvWRKY40 in plant basal immunity, we determined the impact of VvWRKY40 expression on flg22‐induced ROS production. As shown in Figure 5b, VvWRKY40 expression markedly reduced the flg22‐induced ROS production and the protein level of VvWRKY40 in *N. benthamiana* was detected by western blot (Figure 5c). To explore the potential role of VvWRKY40 in plant–pathogen interaction, VvWRKY40 was heterologously expressed in *N. benthamiana* and then the infiltrated area was inoculated with *P. capsici*. The area expressing VvWRKY40 exhibited a larger lesion area (Figure 5d,e). The approach of virus‐induced gene silencing (VIGS) was used to knock down the expression of *WRKY40* in *N. benthamiana* to further investigate the role of WRKY40 in plant immunity. The silencing level was detected by quantitative reverse transcription PCR (RT‐qPCR) and showed that the transcript level of *NbWRKY40* was reduced to 30% (Figure S4a). TRV:*WRKY40* and TRV:Empty plants were inoculated with *P. capsici*. As shown in Figure S4b,c, silencing of *NbWRKY40* significantly reduced *P. capsici* colonization relative to the control. We also did an experiment to investigate the suppression of flg22‐induced ROS production by PvRXLR111 on TRV:*WRKY40* plants in comparison with TRV:Empty plants. However, the results indicated that there was no detectable difference between TRV:*WRKY40* and TRV:Empty plants (Figure S4d). These results suggest that VvWRKY40 could be a negative regulator in plant immunity.

### 2.5 VvWRKY40 is stabilized by PvRXLR111

**FIGURE 6:**
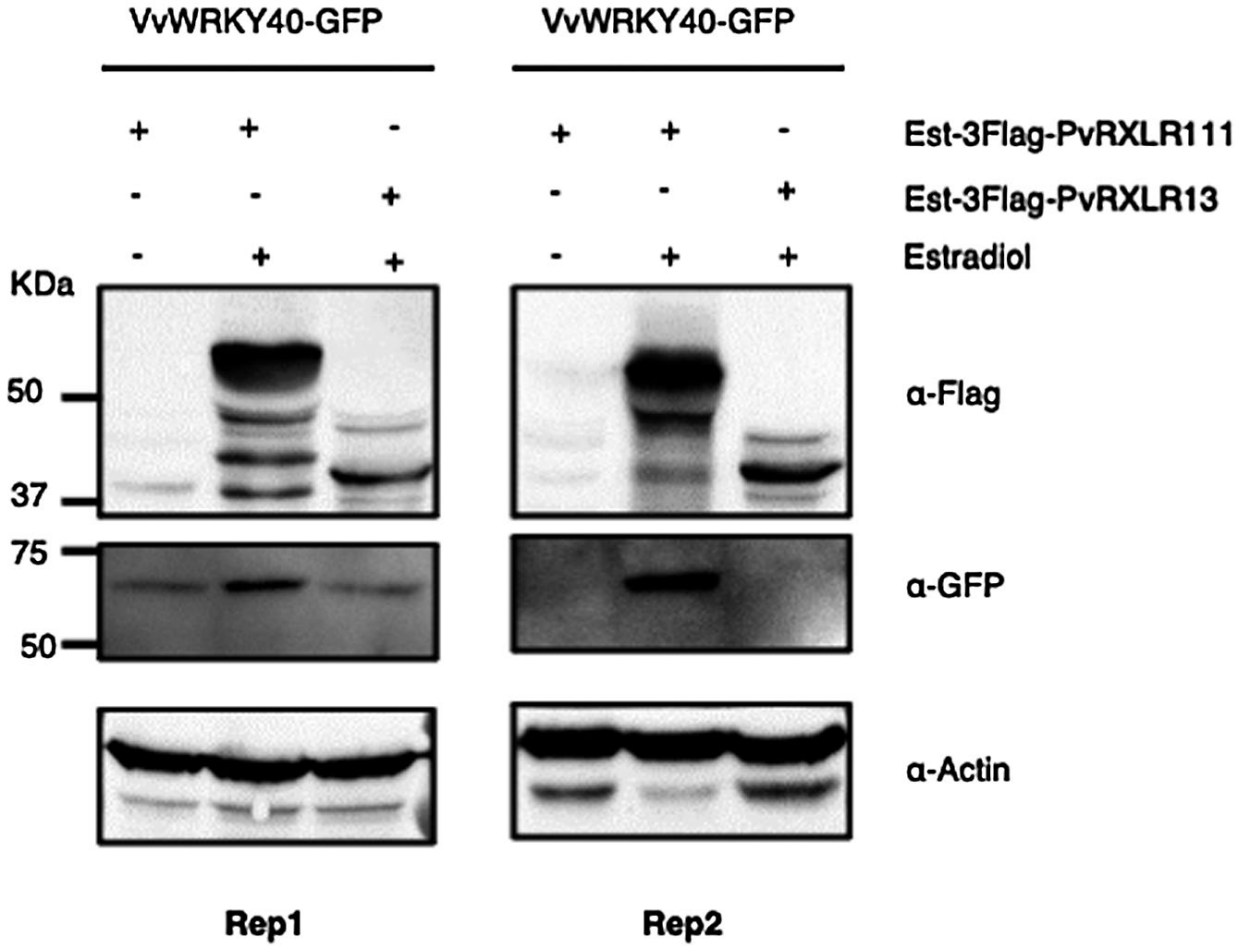
PvRXLR111 stabilizes VvWRKY40. Immunoblot showing an increased signal from VvWRKY40‐GFP in the presence of PvRXLR111‐3FLAG and not with PvRXLR13‐3FLAG. *Agrobacterium tumefaciens* cells containing pER8::*3FLAG‐PvRXLR111* or pER8::*3FLAG‐PvRXLR13* were coinfiltrated in *Nicotiana benthamiana* with pHB::*VvWRKY40‐GFP*, and 24 hr later 10 µM estradiol or DMSO (mock) was injected in the same region. Samples were harvested at 48 hr postinfiltration

Overexpression of VvWRKY40 suppressed flg22‐induced ROS production, which phenocopies the transient expression of PvRXLR111 in *N. benthamiana*, leading us to ask whether PvRXLR111 expression increases the protein level of VvWRKY40. To test this hypothesis, we investigated the protein level of VvWRKY40 when coexpressed with PvRXLR111 or PvRXLR13, an RXLR effector from *P. viticola*, in *N. benthamiana*. *Agrobacterium tumefaciens* containing pER8::*3FLAG*‐*PvRXLR111* or pER8::*3FLAG*‐*PvRXLR13* was coinfiltrated with pHB::*VvWRKY40*‐GFP in *N. benthamiana* and 24 hours later estradiol or dimethyl sulphoxide (DMSO) was injected and samples were harvested at 48 hpi. As shown in Figure 6, there was consistently more VvWRKY40‐GFP in the presence of 3FLAG‐PvRXLR111 than with 3FLAG‐PvRXLR13 or VvWRKY40‐GFP expression alone, suggesting that transient expression PvRXLR111 promotes VvWRKY40 protein accumulation.

## 3 DISCUSSION

### 3.1 PvRXLR111 suppresses plant immunity

Many effectors, including RXLRs, can induce plant cell death, such as PcAvh1 (Chen et al., 2019), RXLR207 (Li et al., 2019a), PpE4 (Huang et al., 2018), Avh241 (Yu et al., 2012), and Avh238 (Yang et al., 2017). On the other hand, some other effectors can suppress the cell death induced by these effectors. For example, Wang et al. (2011) reported that effector Avh172 reversed the cell death caused by effector Avh238 in *N. benthamiana*. Pst DC3000 type III secretion effector AvrPtoB inhibits host programmed cell death induced by HopAD1 (Wei et al., 2015). Further studies revealed that the major function of Avh238 is to suppress plant immunity during pathogen infection. Our results also further support the view that pathogens evolve effectors to suppress plant cell recognition of the cell death‐inducing effectors and cooperation exists between these effectors (Wang et al., 2011). PvRXLR111 induces cell death when expressed in *N. benthamiana*, which is possibly attributed to the recognition of *P. viticola* by *N. benthamiana*, as a nonhost (Liu et al., 2018). Due to the challenge of genetic manipulation of grapevine, it remains unknown whether the recognition machinery also exists in grapevine. Nevertheless, the present studies do show that several PvRXLRs inhibited the cell death induced by PvRXLR111, which raises the possibility that *P. viticola* could evade plant recognition of PvRXLR111. Several lines of evidence in this study support that PvRXLR111 is able to suppress plant immunity: (a) PvRXLR111 suppressed the PTI response triggered by flg22 (Figures 1 and S1); (b) PvRXLR111 suppressed *N. benthamiana* resistance to *P. capsici* in the presence of PvRXLR76 (Figure 2b,c) and increased susceptibility to *H. arabidopsidis* Noco2 (Figure 2d); (c) Pst DC3000 expressing PvRXLR111 displayed enhanced virulence (Figure 2e); (d) PvRXLR111 interacted with, and stabilized, VvWRKY40 (Figures 4 and 6, and Table S1), a negative regulator of plant immunity (Figure 5). These results suggest that PvRXLR111 mainly functions to suppress grapevine immunity during *P. viticola* infection. Future work is needed to obtain genetic evidence supporting this point. In addition to PvRXLR111, several PvRXLRs, including PvAvh74 and PvRXLR16 from *P. viticola*, were also reported to trigger cell death in *N. benthamiana* (Xiang et al., 2017; Yin et al., 2019). It will be interesting to study whether these PvRXLRs have suppressing effects on plant immunity.

To investigate the interference effect of RXLRs on plant immunity, a suppression assay of INF1‐triggered cell death is often performed (Bos et al., 2006; Oh et al., 2009). However, this approach is not suitable for studying cell death‐inducing effectors such as PvRXLR111. In our *N. benthamiana* transient expression system, PvRXLR111 accumulated to a level that sufficiently inhibited flg22‐induced ROS production without affecting cell viability at early time points after agroinfiltration. We thus propose that this is an efficient approach to evaluate the effect of cell death‐inducing effectors on PTI. The phenotype that early PvRXLR111 accumulation inhibits PTI but does not induce cell death also raises the possibility that *P. viticola* may fine tune the level of PvRXLR111 to circumvent host recognition while keeping its inhibitory effect on plant immunity.

### 3.2 Role of VvWRKY40 in plant immunity

Identification of the host targets of effectors is crucial for understanding pathogen virulence and plant susceptibility or resistance. In the present study, we identified VvWRKY40 as a target of PvRXLR111 (Figures 4 and 6, and Table S1), which, to our best of knowledge, is the second host target of PvRXLRs identified so far. Previous studies have revealed that WRKY40 plays a dual role in plant immunity. WRKY40 enhances the resistance of pepper to *Ralstonia solanacearum* and chickpea to *Fusarium oxysporum* (Chakraborty et al., 2019; Dang et al., 2013), while WRKY40 acts as a negative immune regulator in *Arabidopsis* resistance to *Golovinomyces orontii* (Pandey et al., 2010). Our results support the idea that VvWRKY40 is a negative immune regulator because VvWRKY40 expression suppressed the flg22‐induced ROS burst and promoted *P. capsici* infection in *N. benthamiana*. It has been reported that AtWRKY40 negatively regulates the transcription of many signalling components in PTI, including *FLS2*, *BIK1*, and *RBOHD* (Birkenbihl et al., 2017b). It can be suggested that VvWRKY40 functions in a similar fashion to AtWRKY40 to down‐regulate plant immunity.

It has been shown that WRKY40 stability is tightly regulated during biotic and abiotic stress (An et al., 2019; Chakraborty et al., 2019). Further results showed that phosphorylation by CaMPK9 increased the stability of CaWRKY40 during fungal infection (Chakraborty et al., 2019), suggesting that the phosphorylation status of WRKY40 is crucial for its stability. Our results suggest that VvWRKY40 is targeted by PvRXLR111, whose stability is enhanced by PvRXLR111 expression (Figure 6). In the future, it would be interesting to investigate whether the stability of VvWRKY40 is regulated by phosphorylation events affected by PvRXLR111. Our results also show that the expression of *VvWRKY40* was increased during *P. viticola* infection (Figure 5a), indicating that *VvWRKY40* is also regulated at the transcriptional level.

Some similar examples that show how effectors manipulate negative regulators of plant immunity have been reported (Li et al., 2019b; Turnbull et al., 2019; Yang et al., 2016). It may be a common strategy for oomycetes that RXLR effectors use negative regulators of plant immunity to contribute virulence.

We also investigated the contribution of WRKY40 to PvRXLR111‐mediated suppression of flg22‐induced ROS production. The suppression of flg22‐induced ROS production by PvRXLR111 did not show a detectable difference in TRV:*WRKY40* and TRV:Empty plants, and it was observed that PvRXLR111 still strongly suppressed flg22‐induced ROS production (Figure S4d), suggesting two possibilities: (a) the residual transcripts of *NbWRKY40* in TRV:*WRKY40* plants are enough for PvRXLR111 to suppress flg22‐induced ROS production, or (b) some other WRKYs redundantly function in PvRXLR111‐mediated suppression of flg22‐induced ROS production. Indeed, AtWRKY18, AtWRKY40, and AtWRKY60 are functionally redundant in negatively regulating resistance to *P. syringae* (Xu et al., 2006). Furthermore, it has been reported that AtWRKY18 and AtWRKY40 function redundantly as negative regulators of flg22‐induced genes (Birkenbihl et al., 2017b). A similar scenario may also occur in *N. benthamiana*. It is necessary to investigate whether the stability of WRKY18 and WRKY60 are enhanced by PvRXLR111.

**FIGURE 7:**
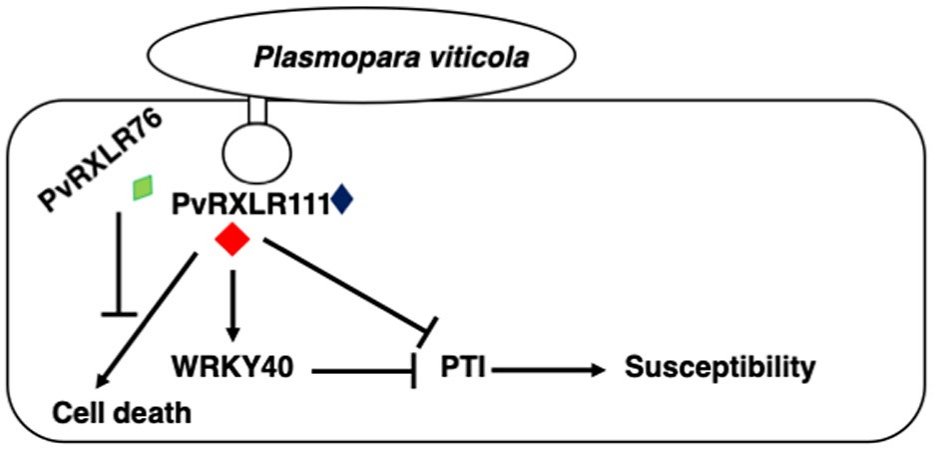
Proposed model for the role of PvRXLR11 in plant immunity. PvRXLR111 induces cell death, while some PvRXLR effectors, indicated by PvRXLR76, suppress PvRXLR111‐induced cell death. PvRXLR111 is also proposed to target and stabilize VvWRKY40, which suppresses PAMP‐triggered immunity (PTI), thereby boosting plant susceptibility

In summary, PvRXLR111 induces cell death, while some other PvRXLR effectors, such as PvRXLR76, could suppress the cell death induced by PvRXLR111 to reinstall the pathogenicity. This fits well in the model of the arms race between pathogen pathogenicity and host plant resistance. Our study demonstrates that effector PvRXLR111 also boosts plant susceptibility through targeting and stabilizing VvWRKY40, a negative regulator of plant immunity, to promote pathogen infection. Although further studies are necessary to fully understand the whole picture about the mechanism of pathogenicity and plant immunity surrounding effector PvRXLR111 and its associates, a mode of action is proposed here based on the findings from the current research (Figure 7).

## 4 EXPERIMENTAL PROCEDURES

### 4.1 Plant materials and culture conditions

*A. thaliana*, *N. benthamiana*, and grapevine (*V. vinifera*) were grown in a greenhouse at 22 °C under white light with a 14 hr light and 10 hr dark cycle.

### 4.2 Vector construction

*PvRXLR111* was cloned from the DNA of *P. viticola* and ligated into the cloning vector pLB for sequencing. The correct sequence of *PvRXLR111* without the signal peptide region was amplified and inserted into PVX (pGR106), pHB‐GFP, and pER8‐FLAG after digestion by appropriate restriction enzymes. To produce pUCP19‐*proAvrPto*:*AvrPto1‐45*‐*His*, the promoter and the first 45 bp of the coding sequence of *AvrPto* were cloned from DNA of Pst DC3000 and inserted into pUCP19‐His. Then, *GFP* or *PvRXLR111* was ligated into pUCP19‐*proAvrPto*:*AvrPto1‐45‐His*.*VvWRKY40* was cloned from the cDNA of *V. vinifera* and the amplified fragments were introduced into pHB‐GFP.

### 4.3 *A. tumefaciens* infiltration assays

The indicated constructs were transformed into *A. tumefaciens* GV3101 by electroporation and the transformants were selected on Luria‐Bertani (LB) medium containing appropriate antibiotics and confirmed by PCR. For infiltration, transformants were cultured for 24 hr at 28 °C 200 rpm and collected and resuspended in infiltration buffer (10 mM MgCl_2_, 10 mM MES pH 5.8, 200 μM acetosyringone) to OD_600_ (0.4). After incubation for 3 hr at 28 °C in the dark, 4‐ to 6‐week‐old *N. benthamiana* leaves were infiltrated with bacterial suspensions using needleless syringes.

### 4.4 Electrolyte leakage assay

Electrolyte leakage assay was done as described in our previous study (Xiang et al., 2017). Electrolyte leakage (%) = 100 × *E*_1_/*E*_2_. To obtain *E*_1_, for each sample six leaf discs of *N. benthamiana* (diameter 1 cm) were incubated in 5 ml of distilled water for 3 hr at room temperature. The conductivity of the bathing solution was measured by a conductivity meter (DDS‐307; Rex Shanghai). Subsequently, the sealed tubes containing bathing solution with leaf discs were boiled for 25 min and cooled to room temperature. The conductivity of the bathing solution was measured to yield *E*_2_. All experiments were repeated three times.

### 4.5 Quantitative reverse transcription PCR

To obtain total RNA, plant material was collected for RNA extraction using an RNA extraction kit (Omega Bio‐Tek Inc.) and the RNA samples were quantified with a NanoDrop spectrophotometer (Thermo Fisher Scientific) and reverse‐transcribed into cDNA using an iScript cDNA synthesis kit (Bio‐Rad Laboratories) according to the manufacturer's instructions. Quantitative PCR was performed on a CFX96 real‐time thermal cycler (Bio‐Rad) with iTaq Universal SYBR Green Supermix (Bio‐Rad Laboratories). The primers used in the assay are listed in Table S2.

### 4.6 ROS production assay

A ROS production assay was done as described as a previous study (Shidore et al., 2017) but modified as follows. *N. benthamiana* leaves were collected 24 or 48 hr after agroinfiltration. The leaf discs (diameter 4 mm) expressing target constructs were incubated in 200 µl of water in a 96‐well plate in the dark for 12 hr and then ROS production of the leaf discs was measured in a detection buffer (100 µM luminol and 10 µg/ml horseradish peroxidase) with or without 1 µM flg22 by a multiplate reader (BioTek) for 55 min.

### 4.7 Callose deposition assays

Callose deposition was detected as described in Xu et al. (2019a). Briefly, discs of *N. benthamiana* were decolored in 95% ethanol and then were washed with 0.07 M phosphate buffer (pH 9.6) and incubated for 1–2 hr in 0.07 M phosphate buffer containing 0.05% aniline blue. Callose deposits were analysed in fields of 1 mm^2^ using ImageJ.

### 4.8 Yeast two‐hybrid assay

A yeast two‐hybrid assay was conducted using the GAL4 system and carried out as described by Xu et al. (2019b). The coding sequences of *VvWRKY40* and *PvRXLR111* without a signal peptide were cloned into pGBKT7 or pGADT7 to generate bait and prey vectors. The indicated construct pairs were cotransformed into AH109 yeast cells and transformed colonies were selected on SD−Leu−Trp medium. The transformants were transferred to SD−Leu−Trp−His medium for interaction analysis.

### 4.9 Pull‐down assay

PvRXLR111‐His, maltose binding protein (MBP), and MBP‐VvWRKY40 recombinant fusion proteins were expressed in *E. coli* BL21 (DE3). The PvRXLR111‐His recombinant protein was purified by Ni‐charged MagBeads (GenScript). The recombinant protein GST‐VvWRKY40 was first incubated with glutathione sepharose 4B beads (GE Healthcare) in lysis buffer without EDTA (50 mM Tris‐HCl, 150 mM NaCl, 0.2% (vol/vol) Triton‐X‐100; pH 7.5) on a rotator at 4 °C for 90 min. The beads were washed three times with lysis buffer and then equal amount of PvRXLR111‐His or GFP‐His was added, respectively. The beads were washed three times with the same buffer after incubation on a rotator at 4 °C for 60 min and boiled in 1.5× SDS loading buffer at 100 °C for 5 min. A western blot was performed using anti‐His antibody (TransGen Biotech Co. Ltd).

### 4.10 BiFC assay

The coding sequences of *VvWRKY40* and *PvRXLR111* without a signal peptide were cloned into pXY106 or pXY104 to generate pXY106‐*VvWRKY40* and pXY104‐*PvRXLR111*. All constructs for BiFC assay were transformed into *A. tumefaciens* GV3101 and combinations were coinfiltrated into *N. benthamiana* leaves. Fluorescence signals were detected with a confocal microscope (Leica TCS SP5II) 40–48 hr after inoculation.

### 4.11 VIGS assay in *N. benthamiana*

For VIGS assays, PCR fragments of NbWRKY40 from *N. benthamiana* cDNA were amplified and inserted into TRV2 vectors by *Kpn*I and *Xma*I. The *Agrobacterium* strains containing pTRV1 vector and pTRV2, pTRV2‐*NbWRKY40*, or pTRV2‐*PDS* were coinfiltrated into two primary leaves of a plant at the four‐leaf stage. The agroinfiltrated plants were then grown for 3–4 weeks before using for *P. capsici* infection and checking gene‐silencing levels (Lan et al., 2019).

### 4.12 VvWRKY40 protein stabilization assay

*A. tumefaciens* cells containing pER8::*3FLAG‐PvRXLR111* or pER8::*3FLAG‐PvRXLR13* were mixed with *A. tumefaciens* cells containing pHB::*VvWRKY40*‐*GFP* and coinfiltrated into *N. benthamiana*. Twenty‐four hours later, estradiol or DMSO was injected in the same region. The leaf discs were collected at 48 hpi for protein extraction. Total protein was extracted with lysis buffer (50 mM Tris‐HCl, 150 mM NaCl, 0.2% (vol/vol) Triton‐X‐100; pH 7.5) and the protein level of VvWRKY40 was detected by western blot using anti‐GFP antibody (TransGen Biotech Co. Ltd).

### 4.13 Pathogen inoculation assays

Pst DC3000 infection was performed as previously described (Hatsugai et al., 2018). Briefly, cell pellets were washed and resuspended at OD_600_ = 0.001 in 10 mM MgCl_2_ and infiltrated into the leaves of 4‐week‐old *Arabidopsis* plants using needleless syringes.

For *H. arabidopsidis* Noco2 infection, 2‐week‐old transgenic lines 24 hr after spray treatment with estradiol or water, were inoculated with Noco2 spores. Five days postinoculation, spores from approximately four seedlings per line were collected and counted by haemocytometer (Li et al., 2010).

*P. capsici* was cultured on oatmeal agar for 7 days at 25 °C. Agar discs (diameter 5 mm) were prepared from the plates with a cork borer and inoculated onto the abaxial surfaces of detached leaves. The inoculated leaves were incubated at 25 °C. The lesions were photographed under UV light and measured at 54 hpi.

*P. viticola* infection was performed as described previously (Lan et al., 2019). The inoculated leaf discs were collected at the indicated time for total RNA extraction.

## Supporting information


**FIGURE S1** PvRXLR111 suppresses flg22‐induced callose deposition. (a) Callose deposition induced by 1 μM flg22 in *Nicotiana benthamiana* transiently expressing PvRXLR111‐GFP or GFP. The representative images were captured at 24 hr after treatment with flg22. Bar = 200 μm. (b) Quantification of callose deposits at 1 hr after treatment with 1 μM flg22 in *N. benthamiana* transiently expressing PvRXLR111‐GFP or GFP. The number of callose spots per 1‐mm^2^ areas was analysed with the ImageJ software. Mean and standard deviations were calculated from three biological replicates (Student’s *t* test, *p < .05)

**FIGURE S2** PvRXLR111‐induced cell death could be suppressed by other PvRXLR effectors. (a) Quantification of PvRXLR111‐induced cell death by electrolyte leakage measurement. *Agrobacterium tumefaciens* cells carrying constructs encoding the indicated PvRXLR effector or GFP with PvRXLR111 were coinfiltrated in *Nicotiana benthamiana*. Mean and standard deviations were calculated from three biological replicates. Different letters indicate significantly different values at *p* < .05 (one‐way analysis of variance, Tukey’s HSD test). (b) Western blot analysis of proteins from *N. benthamiana* leaves transiently coexpressing PvRXLR111‐3FLAG and the indicated PvRXLR effector

**FIGURE S3** Detection of protein expression. (a) Verification of PvRXLR111 expression in transgenic *Arabidopsis* lines 24 hr after spray treatment with estradiol using an anti‐FLAG antibody. (b) Western blot analysis of protein accumulation in bacteria. DC3000 carrying constructs expressing GFP‐His or PvRXLR111‐His were grown in minimal medium

**FIGURE S4** Silencing of *NbWRKY40* attenuates *Phytophthora capsici* colonization. (a) *NbWRKY40* expression levels after virus‐induced gene silencing (VIGS) treatment determined by quantitative reverse transcription PCR. Mean and standard deviations were calculated from three biological replicates (Student’s *t* test, *p < .05). (b) were taken under UV light at 54 hr postinoculation (hpi) and the lesion area was measured at the same time points. (c) Graph showing mean lesion area of *P. capsici* inoculations at 54 hpi on TRV‐infected leaves of each construct. Mean and standard deviations were calculated from three biological replicates (Student’s *t* test, *p < .05). (d) PvRXLR111 inhibits flg22‐induced oxidative burst in *Nicotiana benthamiana* plants silenced for *NbWRKY40*. The reactive oxygen species (ROS) production induced by 1 μM flg22 was determined in leaf discs of *N. benthamiana* silenced for *NbWRKY40* transiently expressing either PvRXLR111‐GFP or GFP at 24 hpi. The results shown are representative of three independent experiments. Each data point consists of six replicates. Error bars indicate standard deviation

**TABLE S1** Plant proteins that associate with PvRXLR111 in planta

**TABLE S2** List of primers used in this study

## Data Availability

The data that support the findings of this study are available from the corresponding author upon reasonable request.
