# Supplementary figures and images for: Plasmopara viticola effector PvRXLR111 stabilizes VvWRKY40 to promote virulence
Source: Mol Plant Pathol. 2020 Nov 30;22(2):231–42. doi: 10.1111/mpp.13020 (PMC7814959; doi:10.1111/mpp.13020)

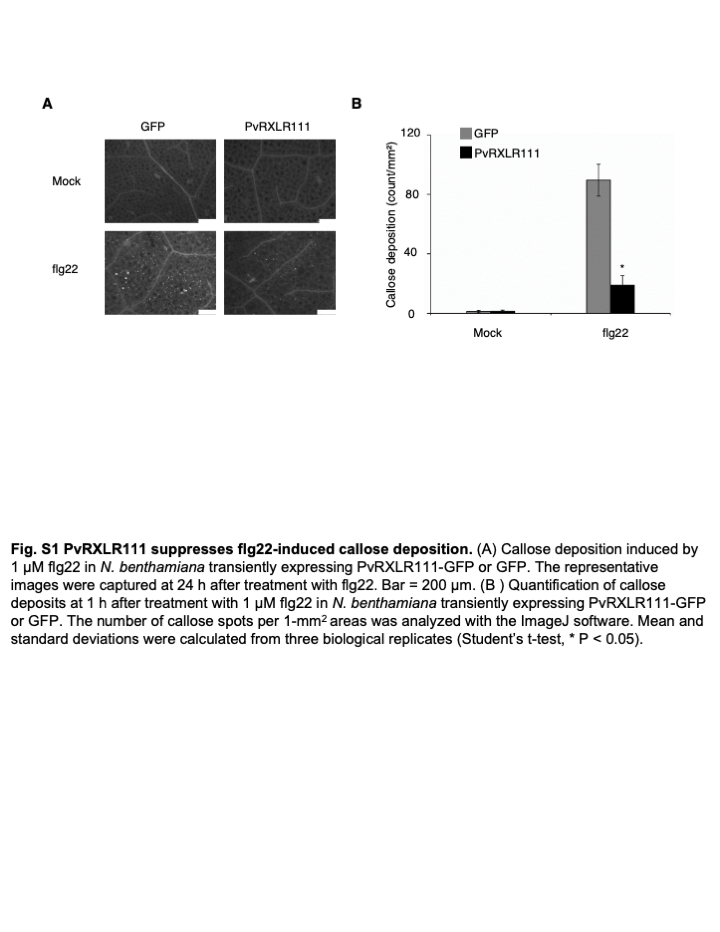

Supplement: Supplementary file 1 — FIGURE S1 PvRXLR111 suppresses flg22‐induced callose deposition. (a) Callose deposition induced by 1 μM flg22 in Nicotiana benthamiana transiently expressing PvRXLR111‐GFP or GFP. The representative images were captured at 24 hr after treatment with flg22. Bar = 200 μm. (b) Quantification of callose deposits at 1 hr after treatment with 1 μM flg22 in N. benthamiana transiently expressing PvRXLR111‐GFP or GFP. The number of callose spots per 1‐mm2 areas was analysed with the ImageJ software. Mean and standard deviations were calculated from three biological replicates (Student’s t test, *p < .05) [file MPP-22-231-s001.tiff]

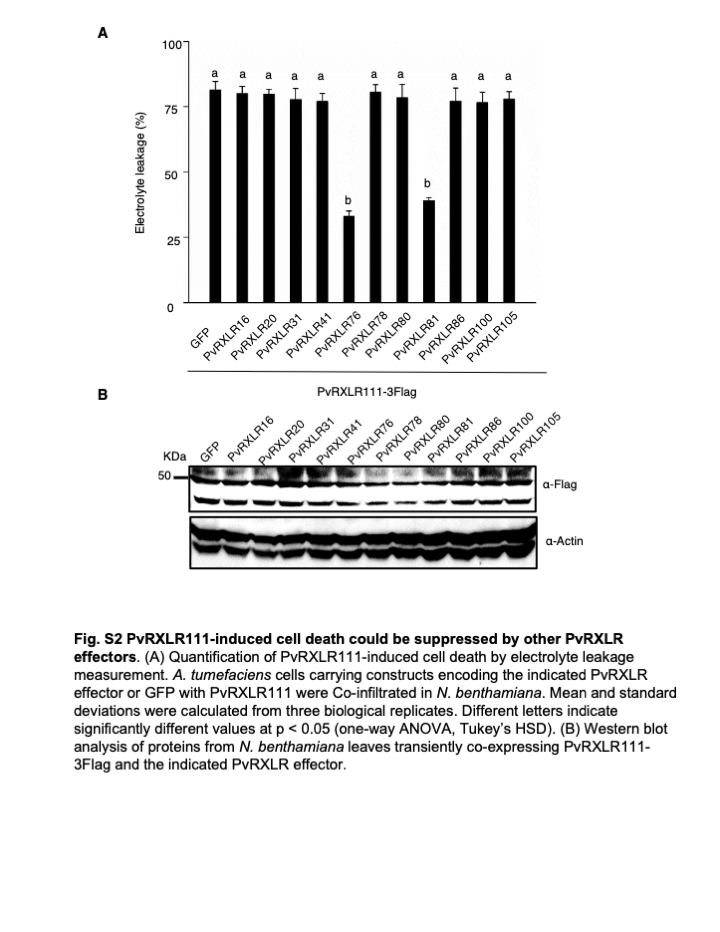

Supplement: Supplementary file 2 — FIGURE S2 PvRXLR111‐induced cell death could be suppressed by other PvRXLR effectors. (a) Quantification of PvRXLR111‐induced cell death by electrolyte leakage measurement. Agrobacterium tumefaciens cells carrying constructs encoding the indicated PvRXLR effector or GFP with PvRXLR111 were coinfiltrated in Nicotiana benthamiana. Mean and standard deviations were calculated from three biological replicates. Different letters indicate significantly different values at p < .05 (one‐way analysis of variance, Tukey’s HSD test). (b) Western blot analysis of proteins from N. benthamiana leaves transiently coexpressing PvRXLR111‐3FLAG and the indicated PvRXLR effector [file MPP-22-231-s002.tiff]

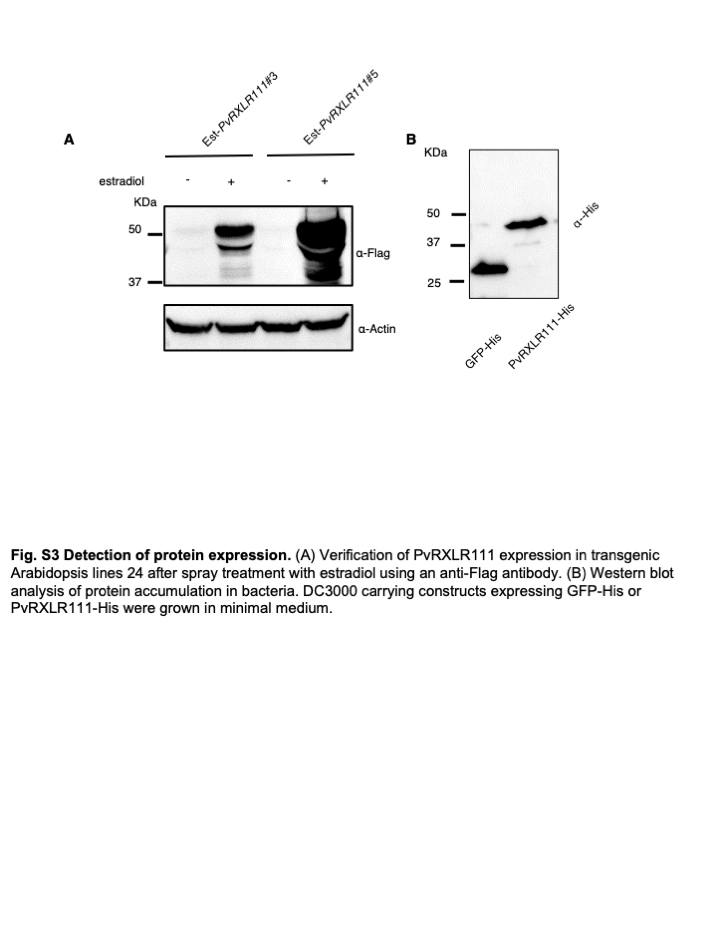

Supplement: Supplementary file 3 — FIGURE S3 Detection of protein expression. (a) Verification of PvRXLR111 expression in transgenic Arabidopsis lines 24 hr after spray treatment with estradiol using an anti‐FLAG antibody. (b) Western blot analysis of protein accumulation in bacteria. DC3000 carrying constructs expressing GFP‐His or PvRXLR111‐His were grown in minimal medium [file MPP-22-231-s003.tiff]

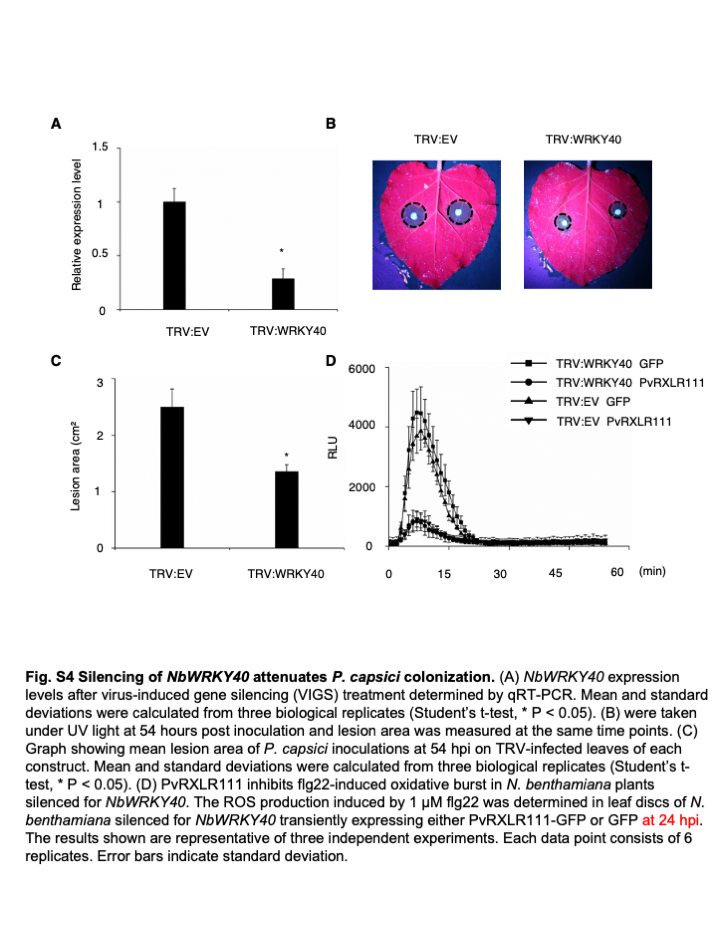

Supplement: Supplementary file 4 — FIGURE S4 Silencing of NbWRKY40 attenuates Phytophthora capsici colonization. (a) NbWRKY40 expression levels after virus‐induced gene silencing (VIGS) treatment determined by quantitative reverse transcription PCR. Mean and standard deviations were calculated from three biological replicates (Student’s t test, *p < .05). (b) were taken under UV light at 54 hr postinoculation (hpi) and the lesion area was measured at the same time points. (c) Graph showing mean lesion area of P. capsici inoculations at 54 hpi on TRV‐infected leaves of each construct. Mean and standard deviations were calculated from three biological replicates (Student’s t test, *p < .05). (d) PvRXLR111 inhibits flg22‐induced oxidative burst in Nicotiana benthamiana plants silenced for NbWRKY40. The reactive oxygen species (ROS) production induced by 1 μM flg22 was determined in leaf discs of N. benthamiana silenced for NbWRKY40 transiently expressing either PvRXLR111‐GFP or GFP at 24 hpi. The results shown are representative of three independent experiments. Each data point consists of six replicates. Error bars indicate standard deviation [file MPP-22-231-s004.tiff]
