# Supplementary material for: Plasmopara viticola effector PvRXLR111 stabilizes VvWRKY40 to promote virulence
Source: Mol Plant Pathol. 2020 Nov 30;22(2):231–42. doi: 10.1111/mpp.13020 (PMC7814959; doi:10.1111/mpp.13020)
Supplement: Supplementary file 5 — TABLE S1 Plant proteins that associate with PvRXLR111 in planta [file MPP-22-231-s005.docx]

**Table S1.** Plant proteins that associate with PvRXLR111 in planta

| Identified protein | Protein score | Accession no. |
| --- | --- | --- |
| SGT1 | 61 | F2VJZ2 |
| SCF ubiquitin ligase | 75 | A0A0K0PU31 |
| UPA17 | 35 | C8YZB0 |
| WRKY40 | 36 | Q9SSX8 |
| Avr9/Cf-9 rapidly elicited protein | 53 | Q9FQZ9 |
| XAP5 | 39 | A0A0M9UMS2 |
| MYC2a transcription factor | 38 | D7P235 |

Sequence accession no. from the UniProt
