# Supplementary material for: Plasmopara viticola effector PvRXLR111 stabilizes VvWRKY40 to promote virulence
Source: Mol Plant Pathol. 2020 Nov 30;22(2):231–42. doi: 10.1111/mpp.13020 (PMC7814959; doi:10.1111/mpp.13020)
Supplement: Supplementary file 6 — TABLE S2 List of primers used in this study [file MPP-22-231-s006.docx]

**Table S2.** List of primers used in this study

| **Cloning** |  |
| --- | --- |
| PvRXLR111-F | ATGCGCGGCACATTAGCGACTG |
| PvRXLR111-R | TCAAGCGTGTTTATTCGACTCG |
| VvWRKY40-F | ATGCCTATGGATAGTTCTAATTG |
| VvWRKY40-R | TCACCATTTTTCAGTTTGATTATG |
| **qRT-PCR** |  |
| Vvactin-F | AGACAGGATGAGCAAGGAAATC |
| Vvactin-R | GCCTCCAATCCATACGCTATAC |
| VvWRKY40RT-F | GAGTCGTTGGGAACTCAGAAA |
| VvWRKY40RT-R | ATGCATCGGTTCGCGTATAG |
| Atactin-F | GTGGATTCCAGCAGCTTCCAT |
| Atactin-R | GCTGAGAGATTCAGATGCCCA |
| Pstoprf-F | AACTGAAAAACACCTTGGGC |
| Pstoprf-R | CCTGGGTTGTTGAAGTGGTA |
